# Effects of *Tremella fuciformis* Mushroom Polysaccharides on Structure, Pasting, and Thermal Properties of Chinese Chestnuts (*Castanea henryi*) Starch Granules under Different Freeze–Thaw Cycles

**DOI:** 10.3390/foods12224118

**Published:** 2023-11-13

**Authors:** Weijing Zhuang, Shuyi Zheng, Feng Chen, Shujuan Gao, Meifang Zhong, Baodong Zheng

**Affiliations:** 1 College of Food Science, Fujian Agriculture and Forestry University, Fuzhou 350002, China; janeyhappy1984@163.com (W.Z.); zshuyi2000@163.com (S.Z.); 18359600595@163.com (S.G.); zhongmeifang2023@163.com (M.Z.); 2 Fujian Provincial Key Laboratory of Quality Science and Processing Technology in Special Starch, Fujian Agriculture and Forestry University, Fuzhou 350002, China; 3 China-Ireland International Cooperation Centre for Food Material Science and Structure Design, Fujian Agriculture and Forestry University, Fuzhou 350002, China; 4 College of Modern Agricultural Technology, Fujian Vocational College of Agriculture, Fuzhou 350119, China; chenfengsf@126.com

**Keywords:** mushroom, polysaccharides, starch, freeze–thaw cycle, structures, physicochemical properties

## Abstract

The purpose of this study was to investigate the effect of *Tremella fuciformis* polysaccharides on the physicochemical properties of freeze–thawed cone chestnut starch. Various aspects, including water content, crystallinity, particle size, gelatinization, retrogradation, thermal properties, rheological properties, and texture, were examined. The results revealed that moderate freezing and thawing processes increased the retrogradation of starch; particle size, viscosity, shear type, hinning degree, and hardness decreased. After adding *Tremella fuciformis* polysaccharide, the particle size, relative crystallinity, and gelatinization temperature decreased, which showed solid characteristics. Consequently, the inclusion of *Tremella fuciformis* polysaccharide effectively countered dehydration caused by freezing and thawing, reduced viscosity, and prevented the retrogradation of frozen–thawed chestnut starch. Moreover, *Tremella fuciformis* polysaccharide played a significant role in enhancing the stability of the frozen–thawed chestnut starch. These findings highlight the potential benefits of incorporating *Tremella fuciformis* polysaccharides in starch-based products subjected to freeze–thaw cycles.

## 1. Introduction

*Castanea henryi* is one of the most important nuts and is widely distributed in East Asia and southern Europe. The output of Chinese *Castanea henryi* accounts for about 83% of the world [1,2]. Due to its unique flavor and nutritional content, it has attracted more attention [3]. Starch is one of the main nutritional components of *Castanea henryi* (40–60% in dry weight) [4], so the structure and physicochemical properties of *Castanea henryi* starch are of great significance to functional properties and application in food.

*Castanea henryi* has more rapidly digestible starch and lower-resistant starch after cooking, which is easy to digest by the human body, causing a high blood sugar response that is not conducive to human health [5,6]. Compared with corn starch, it is easier to retrograde [7]. When starch-based foods are heat-processed, the chains of starch molecules in the food are rearranged and retrograded, which makes the food harder [8]. Therefore, the mutual regulation of the retrogradation properties and digestibility of starch is very important. Starch retrogradation is divided into long-term and short-term. The long-term retrogradation of starch takes place through the recrystallization of the outer short chain of amylopectin, which would shorten the shelf life and harden the texture of starch-based products. Starch foods usually require heat treatment before consumption, and gelatinization occurs during heat treatment. Pasting is an important physical and chemical property of starch and largely determines the nutritional and sensory characteristics of starch foods. When the starch granules undergo irreversible phase changes in excess water, the granules swelling and disruption of their original highly ordered structure occur. A small fraction of starch chain molecules might be cross-linked during cooling and retrogradation [9]. Short-term retrogradation is usually completed within several hours after gelatinization, which is dominated by amylose rearranging in aggregates to form a cross-linked network [10,11]. When the amylose content is more than 50%, it is difficult to gelatinize, and the granules have strong boiling resistance [12]. Short-term retrogradation is an irreversible process of heat; reheating could not restore the state before gelatinization. Therefore, this characteristic could be used to give the product (such as cool skin and rice noodles) strong toughness and surface gloss, as well as a chewy taste [13]. Starch retrogradation is considered a bad property in many cases, such as when bread hardens. At the same time, it also has low enzyme digestibility and glucose release rate, so it is beneficial to health [14]. Thus, the starch regrowth characteristics and digestibility could be slowed down by promoting the formation of short-term regrowth and inhibiting its long-term regrowth [15].

Freezing makes starchy foods easier to store. However, the freeze–thaw treatment usually roughens the surface of starch granules and causes them to be crushed due to the pressure exerted on the double helix structure by water separated from the matrix [16]. During the freezing process, the water molecules in starch gel form ice crystals, and starch molecules aggregate. Upon thawing, the starch gel is separated into water- and starch-rich phases, and repeated freeze–thaw cycles enhance this phase separation and ice growth [17]. Too-low freezing temperatures or repeated freezing would cause starch to form cryogel and sponge textures [18]. The phenomenon is known as dehydration shrinkage [13]. Multiple freeze–thaw cycles lead to irreversible damage to the interaction between the crystallization sequence and the double helix. As the number of freeze–thaw cycles increases, the size of the cavity in the starch gel will also increase, destroying the honeycomb structure and aggravating it. When the number of freeze–thaw cycles was five, the surface of corn starch granules was greatly damaged after freeze–thaw cycles at −20 °C [19]. At the same time, the degree of retrogradation of starch may increase after freezing and thawing, which leads to dehydration, condensation, and the hardening of starch gel.

The stability of frozen–thawed starch paste could be controlled by accelerating the freezing–thawing speed and adding food additives. Accelerating the freezing and thawing speed and adding food additives could be used to control the stability of frozen–thawed starch paste [20]. It is reported that hydrocolloids improve the gelatinization and gel properties of starch [21,22,23]. Xanthan gum could reduce the amount used to form ice crystals, thus improving the freeze–thaw stability of corn starch [24]. Konjac glucomannan could reduce the dehydration shrinkage of starch gel [25]. *Tremella fuciformis* polysaccharide (TP) is the biologically active ingredient of *Tremella fungus*, which is an acidic heteropolysaccharide with α-1,3-mannan as its active center (the side chain is composed of glucose, glucuronic acid, xylose, fucose, arabinose residues, etc.) [26]. *Tremella fuciformis* polysaccharide extract has a certain viscosity, which could replace traditional thickening stabilizers and improve product stability [27]. In addition, *Tremella fuciformis* polysaccharide has a high biological effect and is widely used in the food, cosmetics, and pharmaceutical industries [28].

Controlling the freeze–thaw stability of starch pastes by adding hydrocolloids has been extensively studied, and the texture of starch-based products could be improved by adding small amounts of oligosaccharides and hydrocolloids [29]. Among them, polysaccharides increase viscosity and decrease the level of dehydration [30], and they could also reduce the retrogradation of starch and improve the gel stability of frozen starch [31]. For example, guar gum could promote the structural change of *Castanea henryi* starch so as to effectively inhibit the retrogradation characteristics of *Castanea henryi* starch [32]. The structure of *Tremella fuciformis* polysaccharides may bring unique water retention to starch, which effectively prevents the dehydration of starch during freeze–thawing. However, there is no report on the effect of *Tremella fuciformis* polysaccharides on freeze–thaw stability of starch. At the same time, there are few studies on starch granules, mostly for the study of starch gel, so this paper discusses the effect of *Tremella fuciformis* polysaccharides on freeze–thawed *Castanea henryi* starch to provide theoretical reference for the interaction between *Tremella fuciformis* polysaccharides and *Castanea henryi* starch and the effect of *Tremella fuciformis* polysaccharides on the physical and chemical properties of *Castanea henryi* starch.

## 2. Materials and Methods

### 2.1. Materials

Fresh *Castanea henryis*: Fujian Jianou Mingnong Agricultural Cooperative, white fungus (*Tremella fuciformis*): purchased from local supermarkets.

### 2.2. Preparation of Castanea henryi Starch

Fresh chestnuts were cleaned, and shells were removed. Then mix it with distilled water in a 1:2 ratio. It was ground to a pulp using a pulper (Philips type wall breaker, Jiangsu Philips Co., Ltd., Suzhou, China), and the starch was washed out using a 100-mesh strainer. The remaining filtrate was mixed and then filtered using a 200-mesh strainer. It was placed at 25 °C for 8 h, and the supernatant and brown suspension were discarded. The bottom powder water of the solution was washed with pure water 5 times, and then it was collected and dried in an electrothermal blast drying oven at 40 °C for one day (DGX-80 electric hot blast drying box, Fuzhou Jingke Instrument Manufacturing Co., Ltd., Fuzhou, China). *Castanea henryi* starch could be obtained by sieving with a 120-mesh sieve.

### 2.3. Preparation of Frozen–Thawed Starch

A mixture of *Castanea henryi* starch and *Tremella fuciformis* polysaccharide (CHS + TFP) was prepared as follows: the *Castanea henryi* starch was mixed with *Tremella fuciformis* polysaccharide at a ratio of 7:3. 30 g of *Castanea henryi* starch and 30 g of CHS + TFP were mixed with 30 g of distilled water, respectively (FA2104 electronic analytical balance, Tianjin Fang Rui Instrument Manufacturing Co., Ltd., Tianjin, China), then stirred by magnetic force for 30 min, and it was left for 1 h. Freeze–thaw cycle starch was prepared as follows: the starch was placed in the refrigerator (BC/BD-319HBN, Haier horizontal freezer, Qingdao Haier Special Freezer Co., Ltd., Qingdao, China) at −20 °C for 22 h and then removed and thawed at 25 °C for 2 h; this process was a cycle. The cycles were performed 0, 3, and 10 times (0 CHS, 3 CHS, 3 CHS + TFP, 10 CHS, 10 CHS + TFP), respectively, to form three experiments. At the end of the freeze–thaw cycle, the starch was placed in a refrigerator at −20 °C for 7 days, removed and thawed at 25 °C, and then dried at 50 °C in hot air for spare parts.

### 2.4. Preparation of Tremella fuciformis Polysaccharide

15 g of fresh *Tremella fuciformis* were washed and dried, dissolved in 1:50 g/mL of deionized water for 2–3 h. It was heated in the water bath at 92 °C for 3 h, then cooled to room temperature and centrifuged at 7000 r/min for 20 min. The supernatant was then freeze-dried and ground into *Tremella fuciformis* polysaccharide powder for later use.

### 2.5. Particle Size Distribution Determination

The particle size distribution of CHS-TFP samples was measured using a laser particle size analyzer (Mastersizer 2000, Malvern Instruments, Malvern, UK). Samples were dissolved in deionized water at room temperature for analysis. The refractive index of the dispersant and sample was 1.33 and 1.53, respectively [33]. Each sample was analyzed in triplicate.

### 2.6. Measurement of Fourier Transform Infrared Spectroscopy (FT-IR)

The 10 mg samples are mixed with 1 g of potassium bromide in a quartz mortar. After that, the powders were ground and made into a vacuum mold for pressing into the 0.1 mm^−1^ thin slices. The sample slices were then placed in a Fourier transform infrared spectrometer (70 v, Bruker Optik GmbH, Co., Ltd., Ettlingen, Germany) and analyzed by the light-transmission method. The spectra were collected at wavelengths ranging from 400 to 4000 cm^−1^.

### 2.7. X-ray Diffraction Determination (XRD)

The samples were determined and analyzed with an X-ray diffractometer (Bruker AXS X-ray diffractometer, Bruker GMBH, Karlsruhe, Germany), using the following settings: target Cu Kα radiation (k) = 0.1789 nm, 40 kV tube pressure, 35 mA current, 5–40° scanning measurement range (2θ), and data acquisition step width of 0.05°.

The relative crystallinity (*X_c_*, %) was calculated using the PeakFit software (Ver. 4.12) according to the following equation:

$$X_{c}=\frac{\sum_{i=1}^{n}A_{\mathrm{ci}}}{A_{t}}$$

where *A_ci_* is the area under each crystalline peak with index *i*, and *A_t_* is the total area of the diffraction pattern.

### 2.8. Thermal Properties of Starch (TGA)

Thermal gravimetric analysis (TGA) was performed with a thermogravimetric analyzer (TGA8000 thermogravimetric analyzer, PerkinElmer, Waltham, MA, USA). The three samples were placed in the platinum pan of the TGA furnace, and assessments were accomplished with a heating rate of 10 °C/min from 10 to 800 °C under a nitrogen atmosphere at a flow rate of 20.0 mL/min.

### 2.9. Low-Field Nuclear Magnetic Resonance (LF-NMR)

The transverse relaxation time *T*_2_ was measured using a MesoQMR23-060H-I NMR analyzer (Suzhou Niumag Analytical Instrument Co., Ltd., Suzhou, China) equipped with a 0.5-T permanent magnet corresponding to a proton resonance frequency of 23 MHz at 32 °C. The dried samples were put into cylindrical trays, respectively, and a radio frequency coil with a diameter of 60 mm was placed to collect the attenuation signal from the Carr-Purcell-Meiboom-Gill (CPMG) pulse sequence. The 90° and 180° pulses were respectively at 21 and 41.0 μs, with a *τ*-value (time between 90° and 180° pulses) of 100 μs. Data from 500 echoes were collected as eight scans for repeated use. Decay data were analyzed using MultiExp stock analysis software (3.0, Suzhou Niumag Analytical Instrument Co., Ltd., Suzhou, China).

### 2.10. Determination of Retrogradation

1.00 g of *Castanea henryi* starch and freeze–thaw starch were weighed and prepared into 50 mL of 1% starch solution. After heating to gelation, they were placed in a measuring cylinder and allowed to stand at about 25 °C for precipitation. The volume of supernatant was recorded every 8 min, and the correlation between the rate of condensate water and time was used to reflect the coagulation.

### 2.11. Determination of Pasting Properties of Starch

Firstly, 28 g of 8% starch emulsion solution was prepared, and then the determination conditions of the rapid viscosimeter (Rapid Visco Analyser (RVA) Tech Master Australia Newport Company, Warriewood, Australia) were set. The conditions were set as follows: when the temperature reached 50 °C for 1.0 min. It was heated to 95 °C at a heating rate of 12 °C/min, and the temperature was maintained for 2.5 min. After that, it was cooled to 50 °C at a rate of 12 °C/min and then maintained for 2.0 min. The speed is 960 r/min in the first 10 s and remains at 160 r/min for the other 10 s. The viscosity curve of starch pastes and related experimental parameters were obtained.

### 2.12. Determination of Shear Thinning

#### 2.12.1. Determination of Thixotropy

After the *Castanea henryi* starch and frozen–thawed starch were measured by the rapid viscometer, the starch was completely gelatinized and ready to be measured on the rheometer (MCR301, Anton Paar, Graz, Austria). Firstly, the conical mold is selected with the model CP50-2, and a conical abrasive tool is installed. Then the corresponding measurement procedure is set up. The plate temperature is set to 25 °C, and the shear rate is 0~200 s^−1^. The sample, after gelling, is placed in the central position of the beaker. After extrusion, the sample should be cleaned in time to keep the measuring plate clean and tidy. The correlation between viscosity (*η*) and shear rate (*γ*) was determined. The Herschel–Bulkley model was used to fit the rheological curve.

*τ* = *τ*_0_ + *Kγn*

In the formula, *τ* represents the shear stress, Pa; *τ*_0_ represents the yield stress, Pa; *γ* represents the shear rate, s^−1^; *K* represents the consistency coefficient, Pa·Sn; and *n* represents the fluid index.

#### 2.12.2. Frequency Scanning

The mold model CP50-2 was selected. After installation, the corresponding experimental parameters were set. The appropriate amount of gelatinized sample was placed on the measuring platform of the rheometer at 25 °C. Amplitude scanning was carried out at an angular rate of 10 rad/s, and the appropriate response (*γ*) was selected according to the linear point for testing, and the relationship between storage modulus, loss modulus, and frequency was determined.

### 2.13. Textural Properties (TPA)

The slurry prepared by RVA was transferred to a cylindrical glass mold with a diameter and height of 2 and 1 cm and then refrigerated at 4 °C for 24 h. A texture analyzer (EZ-TEST Shimadzu, Kyoto, Japan) and Trapezium X software software (1.4.5, Shimadzu, Kyoto, Japan) were used for measurement. The starch gel was compressed to 25% of its original height using a cylindrical aluminum probe with a diameter of 40 mm. The samples were continuously pressed twice at a time interval of 10 s, and the texture parameters were obtained from the obtained curves, such as hardness (the maximum force in the first compression cycle), cohesiveness (the ratio of the second peak area to the first peak area), springiness (the time between the end of the first compression cycle and the beginning of the second compression cycle), and gumminess (the product of hardness and cohesion).

### 2.14. Statistical Analysis

All statistical analyses were performed using Origin analysis software (2018), and the data were reported as the mean ± standard deviation of at least three measurements. DPS software was used for significance analysis (*p* < 0.05).

## 3. Results and Discussion

### 3.1. Effects of the Freeze–Thaw Cycle on the Particle Size Distribution of Castanea henryi Starch

**Figure 1:**
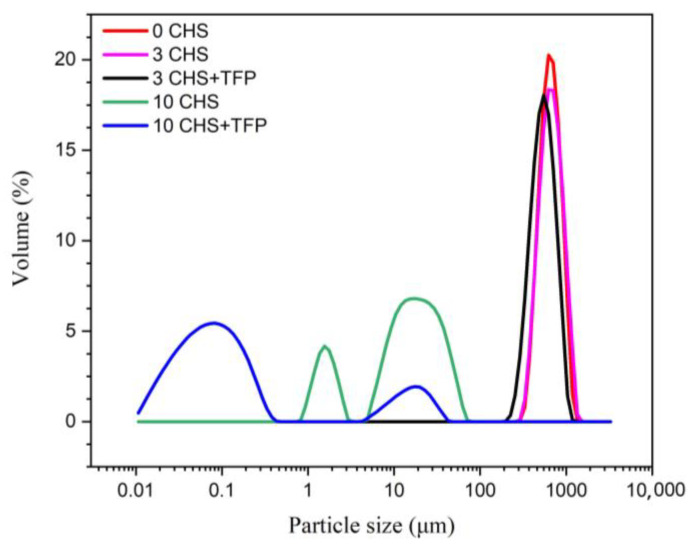
The particle size distribution of *Castanea henryi* starch with different freeze–thaw cycles.

As shown in Figure 1, the average particle size of *Castanea henryi* starch decreased rapidly after undergoing multiple freeze–thaw cycles, particularly evident after 10 cycles. Some starch particles reached nanoscale dimensions. The reduction in particle size could be attributed to the formation of ice crystals and the mechanical forces acting on the starch powder during freezing, causing continuous disruption of the original crystal and helical structures in starch. Consequently, the number of small granule fragments increased, while the starch granules maintained a narrow size distribution and uniformity in particle size. Just like the result of freezing and thawing three times in the figure, which is also related to Guo [34], the uneven particle size distribution may be caused by the volume and surface area effect. The weakening of interactions with molecules within the starch led to the breakage of long-chain molecules [35].

With increasing freeze–thaw cycles, the re-agglomeration and recrystallization of these granules occurred, but the size and distribution became non-uniform due to volume and surface area effects [34]. After undergoing more than 10 freeze–thaw cycles, some starch granules exhibited numerous defects and underwent partial decomposition, with certain starch granule structures being entirely destroyed. As a result, the distribution of particle sizes became bimodal [36]. On the other hand, *Tremella fuciformis* polysaccharides did not significantly alter particle size after three freeze–thaw cycles with starch when compared with starch without *Tremella fuciformis* polysaccharide. However, after 10 cycles of freeze–thaw, a noticeable change occurred, and the particle size became significantly smaller compared to the original size. The phenomenon could be attributed to the dissociation of original starch granules and their interaction with *Tremella fuciformis* polysaccharides, which led to enhanced starch molecule aggregation and a subsequent reduction in particle size compared to native starch.

### 3.2. FT-IR Analysis of Castanea henryi Starch Treated by Different Freeze–Thaw Cycles

**Figure 2:**
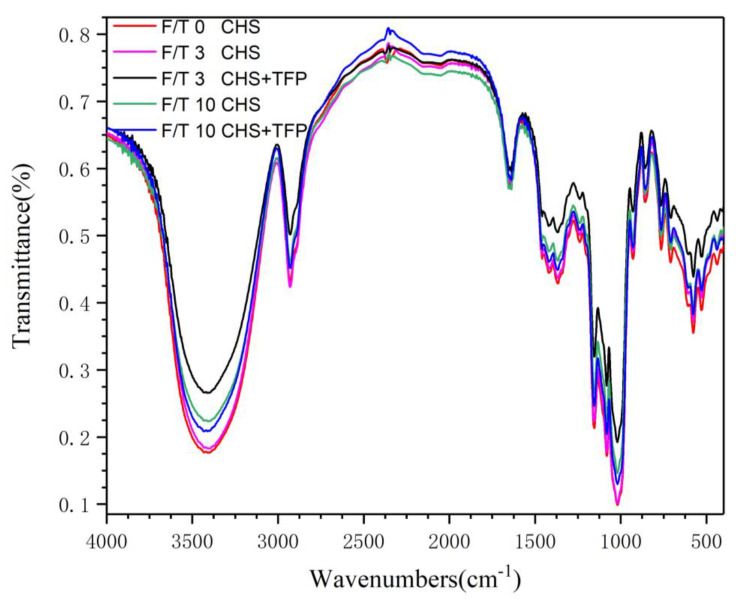
FT-IR of *Castanea henryi* starch with different freeze–thaw cycles.

Infrared spectroscopy is a valuable technique for studying changes in molecular functional groups and conformation [37]. Any modifications in the FTIR spectrum signify changes in the molecular structure of starch, including starch chain conformation, helicity, crystallinity, retrogradation processes, and water content [38]. Figure 2 showed that the infrared spectra of CHS-TFP complexes obtained with different treatments are almost identical, featuring characteristic peaks at 3400 cm^−1^ (O-H stretching vibration), 2930 cm^−1^ (C-H stretching vibration), 1645 cm^−1^ (COO- stretching vibration in a carbohydrate group), 1176 cm^−1^ (C-O, C-C stretching vibration), 1082 cm^−1^ (C-O-H stretching vibration), and 1022 cm^−1^ (C-O stretching vibration), among others. These typical carbohydrate characteristic peaks indicated that freeze–thaw treatment and the addition of polysaccharides did not alter the chemical composition of starch [39].

### 3.3. X-ray Analysis of Castanea henryi Starch Treated by Different Freeze–Thaw Cycles

**Figure 3:**
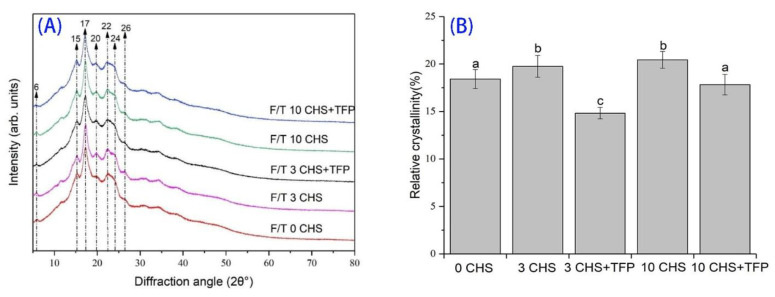
X-ray diffraction pattern (**A**) and relative crystallinity (**B**) of *Castanea henryi* starch treated with different freeze–thaw cycles. The different letters are indicated significant differences.

The X-ray diffraction pattern and relative crystallinity (RS) of freeze–thawed *Castanea henryi* starch-*Tremella fuciformis* polysaccharide with different treatments are shown in Figure 3. A-type starch has two peaks at 17.07° and 18.09° at 2θ and contains nanocrystals with an orthorhombic crystal structure. B-type starch contains nanocrystals with a hexagonal crystal structure, and the peak at 5.63 corresponds to one of the diffraction peaks allowed by the hexagonal crystal structure in the sample. C-type starch has orthorhombic and hexagonal nanocrystals [40]. The freeze–thawed *Castanea henryi* starch prepared in this study showed the structures of C-type starch contain nanocrystals with orthorhombic and hexagonal crystal structures [41]. Compared with the original starch, the relative crystallinity gradually increased with the increase in freeze–thaw cycles, indicating that the amylopectin might be rearranged and bonded, which formed the helix structure through hydrogen bonding [42,43]. Compared with native starch, the significant increase in relative crystallinity of freeze–thaw-treated *Castanea henryi* starch indicated that freeze–thaw treatment might increase the degree of retrogradation of starch and make the structure more orderly. However, the effect of freeze–thaw treatment for 3 and 10 times on crystallinity was not significant (Figure 3). After the addition of *Tremella fuciformis* polysaccharide, the crystal structure of starch changed significantly and significantly reduced the relative crystallinity of starch compared with that without addition, indicating that the starch recrystallization was destroyed to a certain extent during the retrogradation process and inhibited the directional arrangement and retrogradation of *Castanea henryi* starch.

### 3.4. Thermal Properties Analysis (TGA) of Castanea henryi Starch Treated by Different Freeze–Thaw Cycles

Thermogravimetric analysis (TGA) is a technique that investigates the physicochemical properties of substances as the temperature increases. With the increasing temperature, the starch begins to gel. Further temperature increases caused dehydration of the material (involving both free and bound water), which led to decomposition and reorganization. During the process, chemical bonds may also form new volatile and non-volatile products.

**Figure 4:**
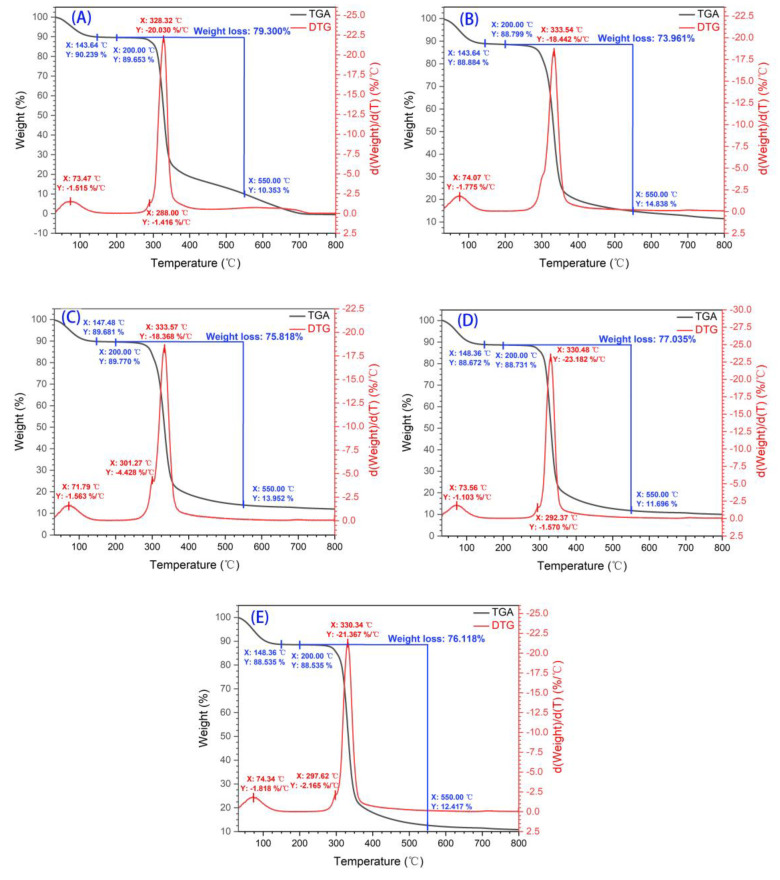
TGA and DTG of *Castanea henryi* starch with different freeze–thaw cycles. (**A**) 0 CHS; (**B**) 3 CHS; (**C**) 3 CHS + TFP; (**D**) 10 CHS; (**E**) 10 CHS + TFP.

The TGA results of *Castanea henryi* starch obtained through different methods at temperatures ranging from 30 °C to 800 °C are shown in Figure 4. The derivative thermogravimetric (DTG) curve indicates that the thermal decomposition of starch mainly occurs in three stages. The first stage, between 70 °C and 290 °C, involves the evaporation of free water. The starch molecule contains numerous hydrophilic groups that could absorb water, which is released as the temperature increases, resulting in a decrease in sample mass. Figure 4 shows that freeze–thaw treatment and the addition of *Tremella fuciformis* polysaccharides increased the loss of free water.

The second stage, between 290 °C and 330 °C, is the result of crystallization, water volatilization, and depolymerization reactions. There is rapid weight loss, which may be associated with the thermal degradation of starch or protein [44]. The rapid weight loss phenomenon is observed in all starches subjected to different treatments. In the third stage, between 330 °C and 420 °C, the remaining material undergoes pyrolysis, decomposing into carbon. During the stage where freeze–thaw treatment leads to weight loss, compared with the original starch, the freeze–thaw treatment would cause a small amount of weight loss. Compared with the starch without *Tremella fuciformis* polysaccharide, the weight loss of *Castanea henryi* starch added with *Tremella fuciformis* polysaccharide is reduced. Particularly, 10 CHS + TFP shows a 5% lower loss rate than the original, indicating that the inclusion of *Tremella fuciformis* polysaccharides enhances the water-holding capacity of starch, making it more resistant to thermal degradation. In addition, the required junction temperature of *Tremella fuciformis* polysaccharide in the second stage is higher than that of the sample without *Tremella fuciformis* polysaccharide, as shown in Figure 4B (245.3 °C) < Figure 4C (301.27 °C) [45,46].

The maximum value of the peak in the DTG curve represented the maximum mass loss rate, and the peak temperature reflected the thermal stability of the polymer. Compared to unfreeze–thawed native starch, freeze–thaw treatment could improve the thermal stability of *Castanea henryi* starch [47].

### 3.5. Moisture Distribution of Castanea henryi Starch with Freeze–Thaw Cycles

LF-NMR (low-field nuclear magnetic resonance) is a technique used to measure the spin relaxation time (*T*_2_), which provides the mobility and states of water molecules in different environments. By fitting and inverting the LF-NMR multi-exponential decay curve, three independent *T*_2_ distribution peaks could be generated, representing different states of water molecules. Smaller *T*_2_ values indicated stronger binding forces and lower mobility in water. Moisture is typically categorized into three forms in food: bound water, fixed water, and free water. Bound water interacts with specific polymer groups; fixed water exists in a polymer network with weak interaction forces; and free water does not bind to any groups. These three types of water correspond to relaxation times *T*_21_, *T*_22_, and *T*_23_, respectively [48].

**Figure 5:**
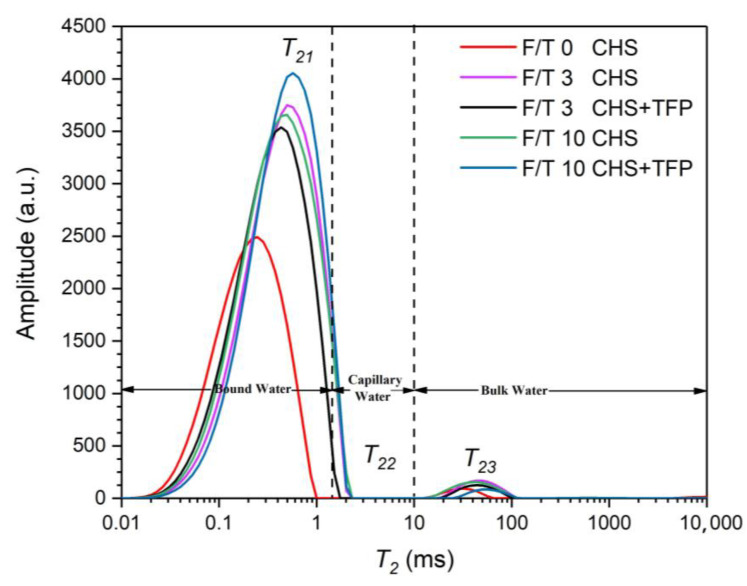
Moisture distribution of *Castanea henryi* starch treated with different freeze–thaw cycles.

The relaxation decay curves of *Castanea henryi* starch extracted using different methods are presented in Figure 5 and Table 1. The decay amplitude is related to the water content. All five extracted starches showed a signal within the range of <1.5 ms (*T*_21_), indicating that the polar groups were tightly bound to crystal water. As the retrogradation time increases, the original single water distribution gradually transforms into a bimodal distribution, indicating the conversion of bound water to free water during the process.

Table 1 shows that the proportion of *T*_21_ (*A*_21_) is over 97%, and it is as high as 99% for the frozen starch thawed three times, indicating that it contains a large amount of tightly bound water. Compared with the original starch, three freeze–thaw cycles increased *T*_21_, which may be due to the reassociation of water and starch molecular chains with the increase of freeze–thaw cycles, leading to the destruction of the original crystallization area of starch and reducing the content of bound water. The addition of *Tremella fuciformis* polysaccharide reduces *T*_21_, which may be due to the reassociation of *Tremella fuciformis* polysaccharide with the starch molecular chain, and a large number of recombined spiral structures would form a denser gel grid arrangement, resulting in a stronger binding force of water molecules in gel. Contrary to *T*_21_, *Tremella fuciformis* polysaccharide starch that has been frozen and thawed for 10 times at this stage contains more free water, which is easy to migrate and vibrate under the influence of a magnetic field, and it is difficult to return to the ground state.

### 3.6. Effects of the Freeze–Thaw Cycle on the Retrogradation Characteristics of Castanea henryi Starch

Starch is susceptible to retrogradation during prolonged storage. Retrogradation occurred when starch was heated and gelatinized, leading to the rearrangement of molecules from the disordered to the ordered state. Bound water is then separated from the starch molecules, causing the starch to settle and enter a retrograded state. Retrogradation is often characterized by the turbidity of starch paste [38].

**Figure 6:**
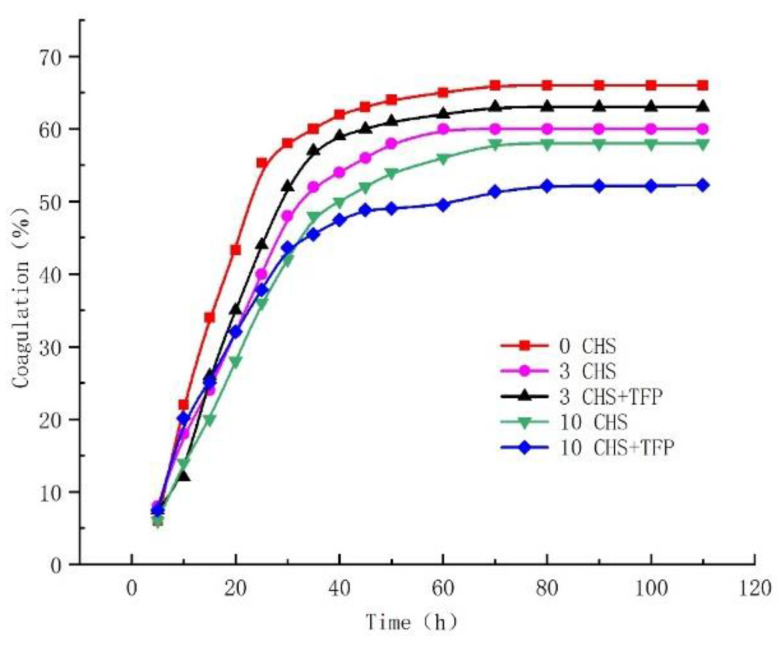
The retrogradation of *Castanea henryi* starch with different freeze–thaw cycles.

Figure 6 shows the formation of precipitates in original *Castanea henryi* starch, freeze–thawed starch, and freeze–thawed starch with added *Tremella fuciformis* polysaccharide after 70 h. The highest water precipitation rates for original *Castanea henryi* starch, starch after 3 freeze–thaw cycles, and starch after 10 freeze–thaw cycles were 65, 60, and 58%, respectively. The highest water separation rates for starch after 3 freeze–thaw cycles and 10 freeze–thaw cycles, with the addition of *Tremella fuciformis* polysaccharide, were 63% and 52.27%, respectively.

The freezing and thawing processes facilitated the dissolution of amylose and the increase in water precipitation after three freeze–thaw cycles with *Tremella fuciformis* polysaccharide, which may be due to the hydrophilic nature of *Tremella fuciformis* polysaccharide. It competes with starch for water in the system and hinders the overflow of amylose [49]. However, after ten freeze–thaw cycles, the addition of *Tremella fuciformis* polysaccharides continues to decrease the water separation rate. It could be attributed to the reorganization of amylose after ten freeze–thaw cycles, leading to the release of some bound water. The addition of *Tremella fuciformis* polysaccharide then combines with the released water, effectively suppressing the dehydration phenomenon.

### 3.7. Rapid Viscosity Analysis (RVA) of Castanea henryi Starch Treated with Different Freeze–Thaw Cycles

During the process of starch gelatinization, water entered the amorphous growth ring. At the same time, the starch granules began to swell, and amylose molecules started to leach out. Only when the amorphous background significantly swelled could sufficient stress be imposed through connecting molecules from amorphous to crystalline regions, initiating the disruption of the crystallites. During this stage, the molecular chains of helix–helices in natural starch might dissociate, followed by helix–helix rearrangement and binding [50,51]. The content of amylose is usually the key factor in determining the rate and degree of starch retrogradation. The longer amylopectin chains could form longer double helices, and higher temperatures are required to fully gelatinize these double helices [52].

**Figure 7:**
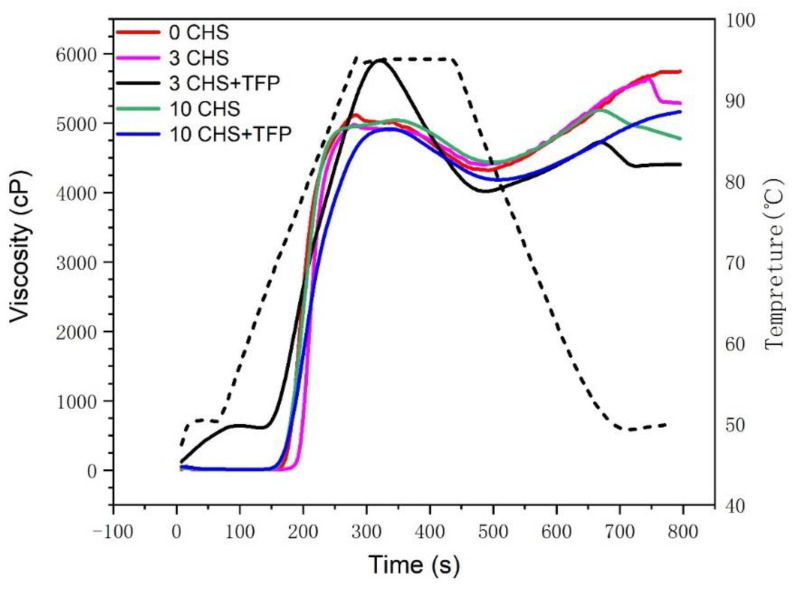
Gelatinization curves of *Castanea henryi* starch treated with different freeze–thaw cycles. The black dashed line (temperature) shows the viscometer heating program.

The gelatinization behavior of freeze–thawed *Chestnut Tremella* starch is shown in Figure 7 and Table 2. Starch is insoluble in water at room temperature but undergoes gelatinization when the temperature exceeds 53 °C. The pasting temperature of starch is the temperature in the RVA curve before the viscosity of starch paste increases substantially. In RVA, it often occurs between 200 and 300 s. After freeze–thaw treatment, the viscosity and initial pasting temperature of the starch system decrease, indicating a low level of retrogradation and reduced retrogradation tendency [53]. The phenomenon could be attributed to the disruption of covalent bonds by freezing and thawing, which leads to a change in the molecular structure of amylose, making the amorphous regions looser and less stable. *Tremella fuciformis* polysaccharides have been shown to increase the viscosity of the system, likely due to their accumulation on the surface of starch granules, resulting in changes in hydrogen bonding on the surface [54].

The parameters *BD* (degree of disintegration) and *SB* (degree of association) reflect the disintegration and association of starch during pasting, respectively, and are important indicators for the decomposition of granules and chains during the process [55]. The decrease in *PV* and *BD* at 3 CHS compared to 0 CHS showed that freeze–thawing treatment would help starch recombination and improve the stability of starch paste against shear. The increase in *BD* at 3 CHS + TFP compared to 3 CHS may be due to the fact that *Tremella polysaccharide* polysaccharides improve the solubilization capacity of cone chestnut amylose-polysaccharides, but solubilized starch paste becomes fragile and is more susceptible to mechanical shear damage, and it also improves starch retrogradation [56]. This is also consistent with the results of Zhang’s study [56]. Moreover, the interaction between *Tremella fuciformis* polysaccharides and amylopectin promoted the gelatinization of starch, and the freeze–thaw treatment induced partial destruction of the internal chain arrangement of amylopectin [57]. The disruption was beneficial for the interaction between starch and *Tremella fuciformis* polysaccharides, leading to further improvements in the properties of the starch system.

### 3.8. Effect of Adding Tremella polysaccharide on the Rheological Properties of Castanea henryi Starch after the Freeze–Thaw Cycle

#### 3.8.1. Shear Thinning Characteristics

**Figure 8:**
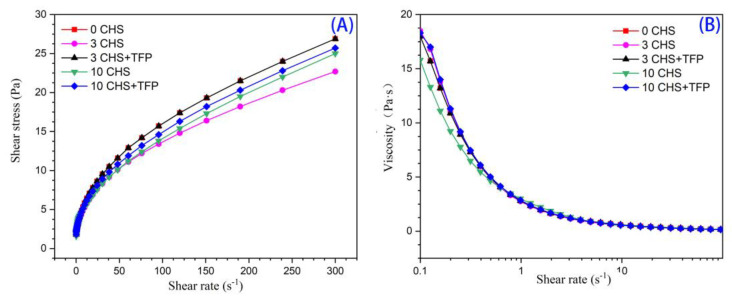
Steady flow curves of *Castanea henryi* starch. (**A**) Shear stress. (**B**) Viscosity.

Figure 8 shows the flow behavior of frozen–thawed *Castanea henryi* starch paste under steady shear. As the shear rate increased, the shear stress was also increasing with shear-rate flow characteristics. The experimental data for flow curves were fitted to a power law model (Table 3), resulting in high regression coefficients (*R*^2^ ≥ 0.98). The flow behavior indices (*n*) ranging from 0.39 to 0.42 were all less than 1.0, indicating that the starch exhibits pseudoplastic and shear-thinning behavior.

The *K* value represents the consistency size [58], decreasing significantly after freezing and thawing and the addition of *Tremella fuciformis* polysaccharides, which showed that freezing and thawing cause damage to the starch structure and reduce the viscosity of the sample, while the incorporation of *Tremella fuciformis* polysaccharides inhibits the leaching of amylose, resulting in a decrease in the consistency coefficient [59].

The apparent viscosity decreased with the increasing shear rate, with a sharp drop in the early stage, indicating the presence of shear thinning behavior characteristic of pseudoplastic fluids [60]. With the increase in freeze–thaw cycles, the breaking of intermolecular and intramolecular hydrogen bonds leads to a looser structure, resulting in reduced viscous resistance, apparent viscosity, and degree of shear thinning [61].

At 10 freeze–thaw cycles, the dense structure of the crystallization zone is destroyed, causing a more significant decrease in the curve. However, the addition of *Tremella fuciformis* polysaccharides increased the apparent viscosity, suggesting that it may form a more stable network structure and increase flow resistance [62].

#### 3.8.2. Dynamic Frequency Scanning

**Figure 9:**
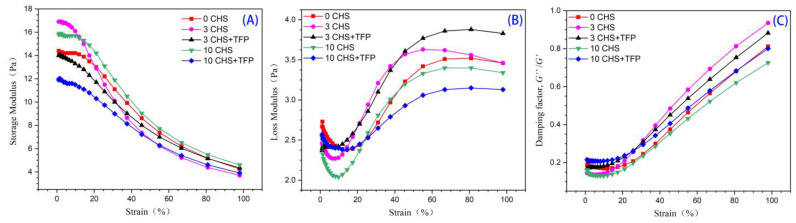
Amplitude sweeps of *Castanea henryi* starch treated by different freeze–thaw cycles. (**A**) Storage modulus (*G*′). (**B**) Loss modulus (*G*″). (**C**) Damping factor (*G*″/*G*′).

The viscoelasticity of starch paste could be characterized by the storage modulus (*G*′) and loss modulus (*G*″) obtained from shear strain scanning. Figure 9 showed that the storage modulus (*G*′) of native starch is higher than the loss modulus (*G*″), indicating that the starch paste exhibits solid characteristics [63].

After freezing and thawing, the storage modulus increased, suggesting that the treatment may lead to the formation of a more stable system compared to the original starch. It was shown that the treatment may form a more ordered system, possibly due to the double helix structure of the starch side chain rearrangement. This result is also consistent with the previous X-ray and infrared results, indicating that freeze–thaw could increase the order of starch and, at the same time, aggravate the retrogradation of starch.

Figure 9 also revealed that the yield strain of starch decreased after freezing and thawing, and the addition of *Tremella fuciformis* polysaccharides intensified the effect. It indicated that the starch with *Tremella fuciformis* polysaccharides had undergone 10 freeze–thaw cycles and exhibited a higher damping coefficient. A sample with a high damping coefficient displayed a more pronounced viscous behavior, resembling a flowing liquid. On the other hand, materials with lower damping coefficients exhibited a more pronounced elastic response to applied loads, behaving more like a solid with reduced flow. Therefore, more deformation energy was stored as potential energy within the material, making it more stable [64].

### 3.9. The Effect of Adding Tremella fuciformis Crude Polysaccharide on the Texture of Chestnut Starch after Freeze–Thaw Cycles

Table 4 shows the texture parameters of starch obtained from TPA measurements. Hardness is the maximum force recorded in the first compression cycle of TPA [65], which is due to the swelling capacity of starch granules. Compared with 3 CHS, it could be found that 10 CHS and 3 CHS + TFP reduced and increased the swelling capacity of starch, respectively, so their swelling capacity decreased and increased, respectively. Cohesiveness is related to the interaction within starch [66]. Compared with 0 CHS, 3 CHS, and 10 CHS, the cohesiveness decreased and increased, respectively, which may be due to the failure of starch structures to form a new network structure after three freeze–thaw cycles, but the network structure formed again after 10 freeze–thaw cycles. The addition of *Tremella fuciformis* could increase the gel properties of starch, including hardness, springiness, and cohesiveness. Both freezing and thawing and the addition of *Tremella fuciformis* polysaccharides improved the gelatinization of the starch paste of *Castanea henryi*. The addition of *Tremella fuciformis* polysaccharides induced a more significant change in the gel recovery, possibly due to the interaction between amylopectin and *Tremella fuciformis* polysaccharides. Studies have shown that the addition of *Tremella fuciformis* polysaccharides enhances the texture properties of ordinary starch and even maintains a smooth surface of the starch gel [67]. The gel formation involved the interaction between starch pastes, and the freeze–thaw treatment and the addition of *Tremella fuciformis* polysaccharide effectively expanded the starch granules, leading to the leaching of soluble starch chains into paste. These dissolved starch chains become entangled and embedded in a continuous gel matrix, thereby reinforcing the gel network [56].

## 4. Conclusions

With the increase in the number of freeze–thaw cycles, the structure of *Castanea henryi* straight-chain starch might be disrupted, and the water precipitation rate might decrease. The freeze–thaw cycle could make the starch more easily retrograded. The viscous resistance as well as the apparent viscosity and shear thinning degree are reduced, causing mechanical damage to *Castanea henryi* starch, while the ice crystal expansion caused by freezing would lead to a reduction in the swelling rate of starch granules. However, the addition of *Tremella fuciformis* polysaccharide could decrease the particle size, relative crystallinity, and gelatinization temperature, which made the chinquapin starch present solid characteristics.

These results showed that the interaction between *Tremella fuciformis* polysaccharide and starch could improve the freeze–thaw stability of *Castanea henryi* starch. *Tremella fuciformis* polysaccharide could effectively improve the water-holding capacity of starch. At the same time, it could also inhibit the destruction of starch structure by freezing and retrogradation during freeze–thaw cycles. This study could provide a theoretical basis for the application of *Tremella fuciformis* polysaccharide in *Castanea henryi* starch.

## Figures and Tables

**Table 1:** Moisture distribution of *Castanea henryi* starch with different freeze–thaw cycles.

Sample	*T*_21_ (ms)	*A*_21_ (%)	*T*_22_ (ms)	*A*_22_ (%)	*T*_23_ (ms)	*A*_23_ (%)
0 CHS	0.25	98.15	32.75	1.70	10,000	0.14
3 CHS	0.50	97.70	43.29	2.30		
3 CHS + TFP	0.43	98.28	43.29	1.73		
10 CHS	0.50	97.57	43.29	2.35	613.59	0.08
10 CHS + TFP	0.57	99.04	57.22	0.96		

**Table 2:** Gelatinization characteristics of *Castanea henryi* starch treated by different freeze–thaw cycles. The different letters are indicated significant differences.

Sample	Pasting Temperature (°C)	PV (cP)	BD (cP)	SB (cP)
0 CHS	80.40 ± 7.09 ^a^	5117.01 ± 396.33 ^a^	795.90 ± 20.01 ^a^	897.11 ± 43.62 ^a^
3 CHS	82.81 ± 9.15 ^a^	4977.90 ± 171.47 ^a^	568.63 ± 19.12 ^b^	874.14 ± 44.73 ^a^
3 CHS+TFP	76.40 ± 2.27 ^b^	5902.21 ± 340.92 ^b^	1884.52 ± 144.55 ^c^	711.53 ± 32.64 ^b^
10 CHS	74.11 ± 3.31 ^b^	5041.21 ± 598.28 ^a^	605.04 ± 44.51 ^b^	752.59 ± 31.88 ^c^
10 CHS + TFP	75.62 ± 4.60 ^b^	4916.42 ± 333.88 ^a^	733.36 ± 61.70 ^c^	539.09 ± 28.83 ^d^

*PV*: peak viscosity; *BD*: breakdown; *SB*: setback.

**Table 3:** Power-law parameters of *Castanea henryi* starch treated with different freeze–thaw cycles.

Sample	*K*	*n*	*R* ^2^
0 CHS	2.33	0.42	0.99
3 CHS	2.27	0.40	0.99
3 CHS + TFP	2.33	0.42	0.99
10 CHS	2.15	0.42	0.99
10 CHS + TFP	2.26	0.42	0.99

**Table 4:** TPA characteristic values of *Castanea henryi* starch treated with different freeze–thaw cycles.

Sample	Hardness/g	Springiness	Cohesiveness	Gumminess	Resilience
0 CHS	169.25 ± 2.00 ^d^	1.10 ± 0.22 ^b^	0.76 ± 0.04 ^a^	127.75 ± 4.20 ^c^	0.52 ± 0.04 ^c^
3 CHS	148.38 ± 2.10 ^b^	0.91 ± 0.05 ^a^	0.72 ± 0.08 ^a^	116.06 ± 5.70 ^b^	0.40 ± 0.02 ^a^
3 CHS + TFP	152.74 ± 3.21 ^c^	1.09 ± 0.01 ^b^	0.83 ± 0.03 ^b^	122.11 ± 2.42 ^c^	0.45 ± 0.02 ^b^
10 CHS	139.22 ± 1.18 ^a^	1.23 ± 0.24 ^c^	0.85 ± 0.05 ^b^	109.82 ± 3.45 ^a^	0.61 ± 0.04 ^d^
10 CHS + TFP	154.50 ± 4.18 ^c^	1.39 ± 0.10 ^d^	0.99 ± 0.06 ^c^	126.89 ± 4.36 ^c^	0.70 ± 0.08 ^e^

The different letters were significantly different (*p* < 0.05).

## Data Availability

The data used to support the findings of this study can be made available by the corresponding author upon request.
